# Supplementary material for: Rutin Promotes Pancreatic Cancer Cell Apoptosis by Upregulating miRNA-877-3p Expression
Source: Molecules. 2022 Mar 31;27(7):2293. doi: 10.3390/molecules27072293 (PMC9000526; doi:10.3390/molecules27072293)
Supplement: Supplementary file 1 [file molecules-27-02293-s001.zip › molecules-1594527-supplementary.pdf]

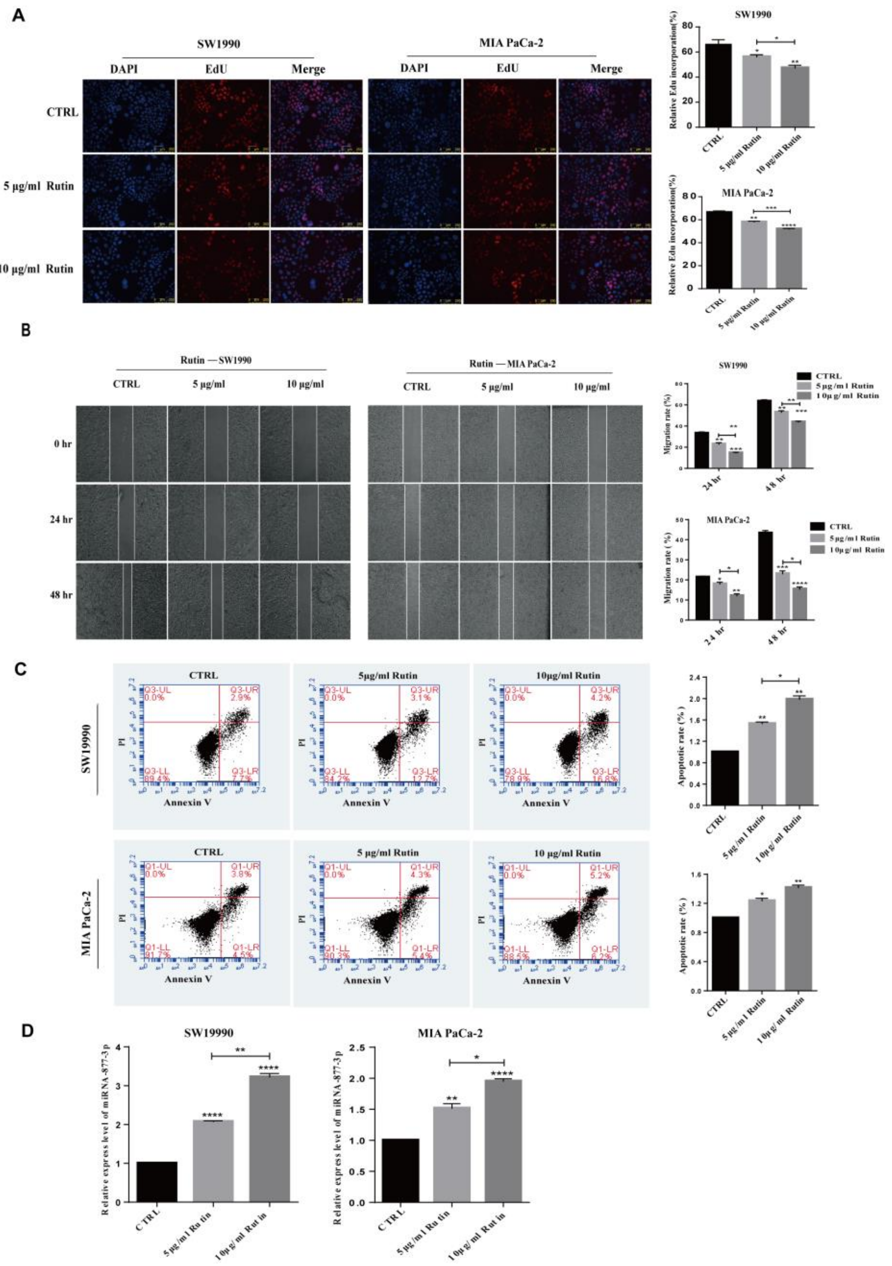

Figure S1. Rutin affects the SW1990 and MIA PaCa-2 cells behavior. A, EdU assays revealed that SW1990 and MIA PaCa-2 cells proliferation were significantly suppressed after rutin treatment for 24 h or 48 h. B, Wound healing assay revealed that rutin treatment for 24 h or 48 h significantly repressed the migration ability of SW1990 and MIA PaCa-2 cells. C, Flow cytometric analysis showed that SW1990 and MIA PaCa-2 cells apoptosis were enhanced by rutin administration for 48 h. D, qRT-PCR analysis verified that rutin could significantly upregulate the miRNA-877-3p expression in SW1990 and MIA PaCa-2 cells. *$p < 0.05$, **$p < 0.01$, ***$p < 0.001$.

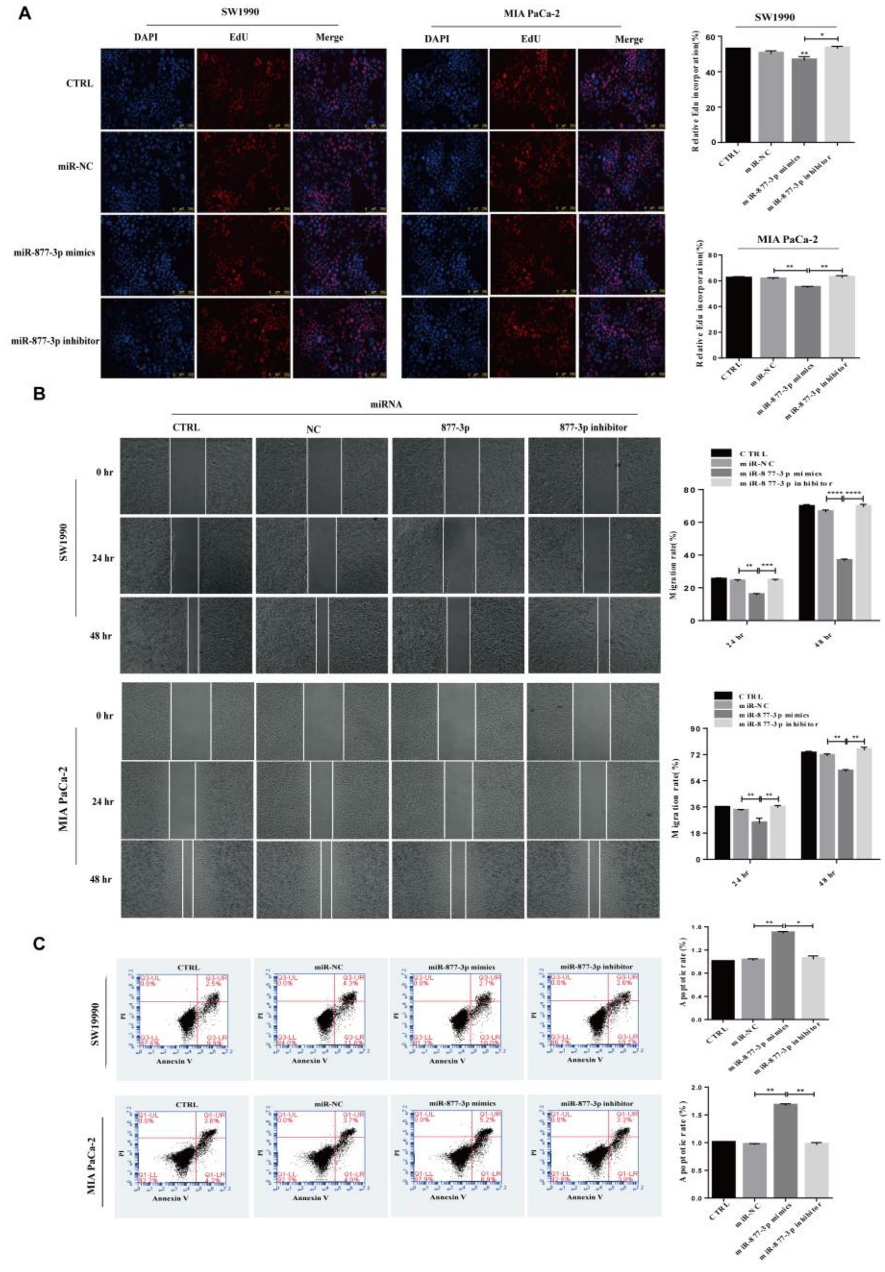

Figure S2. miRNA-877-3p suppressed cells proliferation, migration and promoted cells apoptosis in SW1990 and MIA PaCa-2 cells. A, EdU assays revealed that miRNA-877-3p mimcs suppressed cells proliferation and miRNA-877-3p inhibitor promoted cells proliferation at 48 h after transfection into SW1990 and MIA PaCa-2 cells. B, Wound healing assay showed that miRNA-877-3p mimcs suppressed cells migration and miRNA-877-3p inhibitor promoted cells migration of SW1990 and MIA PaCa-2 cells. C, Flow cytometric assay showed that miR-877-3p mimics promoted PANC-1 cell apoptosis of SW1990 and MIA PaCa-2 cells. *$p < 0.05$, **$p < 0.01$, ***$p < 0.001$.

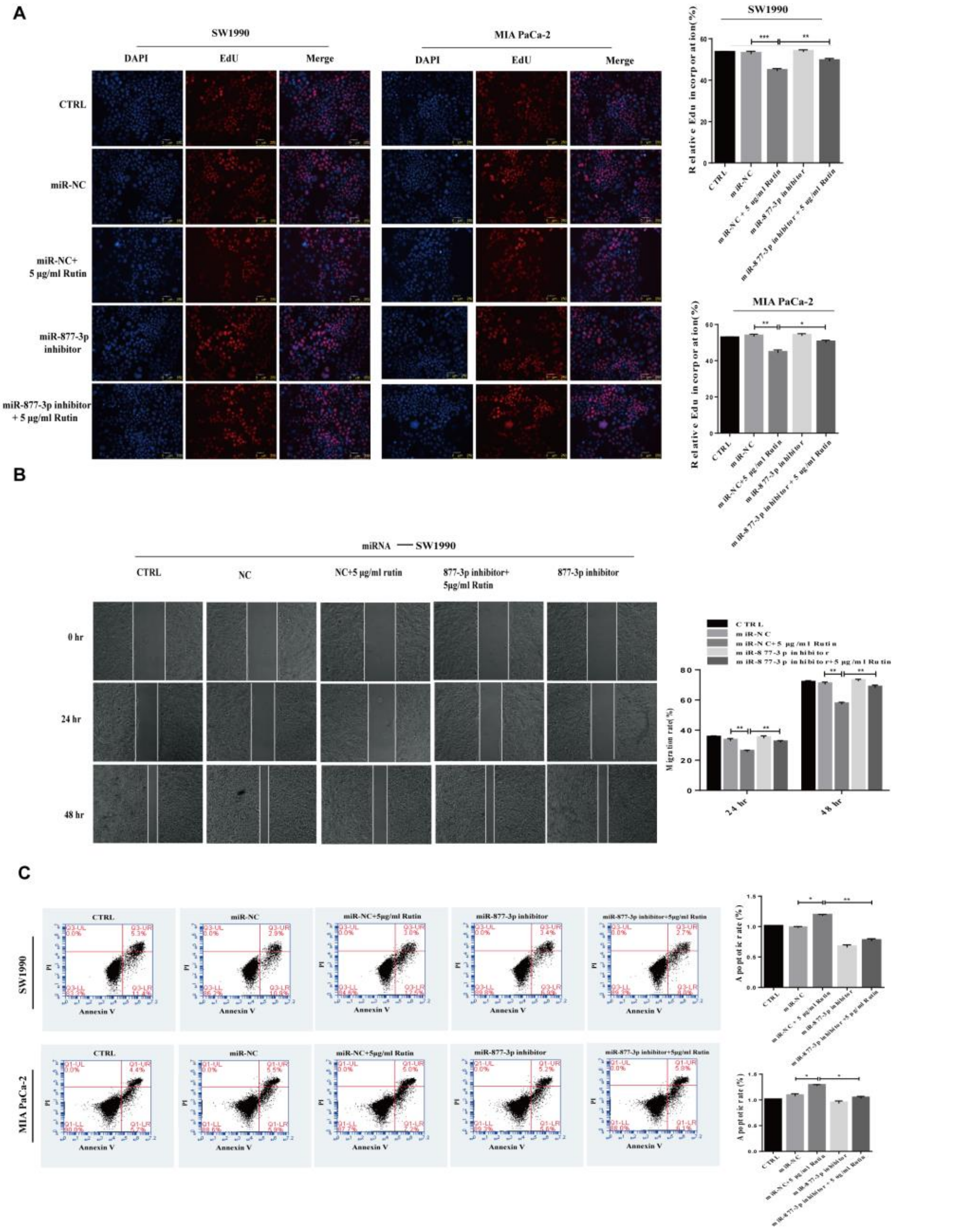

Figure S3. Rutin affected the behavior of the cells by upregulating miR-877-3p expression in SW1990 and MIA PaCa-2 cells. A, Downregulated miR-877-3p effectively reversed the effect of rutin on proliferation of SW1990 and MIA PaCa-2 cells. B, Rutin significantly inhibited SW1990 cells migration by up-regulating miR-877-3p expression. C, Downregulation of miR-877-3p could reverse rutin's pro-apoptotic effect in SW1990 and MIA PaCa-2 cells. **p* < 0.05, ***p* < 0.01, ****p* < 0.001.
